# Use of complementary and alternative medicine in pregnancy: a cross-sectional survey on Iraqi women

**DOI:** 10.1186/s12906-016-1167-0

**Published:** 2016-07-07

**Authors:** Jung Hye Hwang, Yu-Rim Kim, Mansoor Ahmed, Soojeung Choi, Nihad Qasim Al-Hammadi, Nameer Muhammad Widad, Dongwoon Han

**Affiliations:** Department of Obstetrics and Gynecology, University of Hanyang College of Medicine, Seoul, South Korea; Institute of Health Services Management, Hanyang University, Seoul, South Korea; Department of Global Health and Development and Department of Preventive Medicine, Hanyang University, College of Medicine, 222 Wangsimni-ro, Seongdong-gu, Seoul 04763 South Korea; Basrah Health Directorate, Ministry of Health, Basrah, Iraq

**Keywords:** Pregnant women, Iraq, Complementary and alternative medicine

## Abstract

**Background:**

Due to the lack of strong evidence on safety and efficacy of complementary and alternative medicine (CAM) approaches, the use of CAM in women during pregnancy could be hazardous for mother and fetus. Meanwhile, little is known regarding the patterns, the reasons and the factors affecting use of CAM among pregnant women in Iraq.

**Methods:**

A cross sectional survey design was used to carry out face-to-face interviews with 335 consecutive pregnant women. The questionnaire comprised of three sections: socio-demographic characteristics, pregnancy-related aspects and the patterns and attitudes towards use of CAM. Determinants of CAM use were assessed through the logistic regression analysis.

**Results:**

Three hundred thirty-five pregnant women completed the questionnaire. 56.7 % reported using at least one form of CAM modalities. In total, 24 different types of CAM were used; with herbal medicine (53.7 %) and multivitamins (36.3 %) the most commonly used modalities. From the logistic regression analysis, the variables positively associated with CAM use were: rural residence (odds ratio (OR) 2.0, *p* < 0.01), no occupation (OR 2.7, *p* < 0.05), high income (OR 2.0, *p* < 0.05), perceived healthy status (OR 2.6, *p* < 0.05) and ever use of contraception (OR 2.0, *p* < 0.01). Only 0.5 % of CAM users disclosed their CAM use to physicians.

**Conclusions:**

The proportion of CAM users among pregnant women is relatively high and it is important to learn what types of CAM they use. However, disclosure of CAM use was extraordinarily low. Given the low rate of disclosure, it should be ensured that physicians establish good level of communication with pregnant women and have adequate knowledge of CAM.

## Background

The prevalence of complementary and alternative medicine (CAM) use in pregnant women is increasing and is widespread in developed and developing countries [1–3]. These women take a wide range of CAM such as herbs, vitamins and minerals, massage, aromatherapy, acupuncture, homeopathic remedies and Reiki as well as psychological, physical and spiritual techniques [4–6]. However, given the global initiative concerning evidence based medicine and the lack of robust data on safety and efficacy of CAM approaches, health professionals and policymakers have become increasingly concerned about the use of CAM by women during pregnancy [7].

Recent studies reveal that over one third of pregnant women in the USA used one or more CAM therapies during previous year [8, 9]. In the UK, 57.1 % of women reported their CAM use during pregnancy [6]. Surveys from Middle East countries have reported that prevalence of CAM use during pregnancy is 40.0 % in Palestine [10], about 75 % in Jordan [11], and 22.3 % in Iran [12]. However, little is known about the extent to which CAM is used by women during pregnancy in Iraq. It is important to obtain history on CAM use at any time but particularly in pregnancy, in order to provide proper counselling to the expecting mothers. Some of the CAM may have unrecognized effects on pregnancy or labor, have interactions with prescribed medications and have potentially serious complications on fetus.

Iraq, as an Arabian Gulf state, has witnessed a rapid socio-economic transition over the last two decades [13]. Since then, the Iraqi health care system has been seriously affected as a result of different wars, internal conflicts, international sanctions and political instability [14, 15]. Under Iraqi health delivery system, which has been on a centralized, curative and hospital-oriented model, maternity services are provided by public institutions and obstetricians’ private clinics that are widely distributed mainly in urban areas [16]. These events resulted in a substantial fall in major health indices and left a crippled health system struggling to meet population needs [14, 17]. The maternity care services in particular did not escape these damaging effects and continue to suffer from problems common throughout the health care system [18]. The antenatal care services in Iraq suffer from challenges common to the primary health care system [19]. These challenges are mainly related to inappropriate health service delivery including irrational use of health services, poor referral system, poor infrastructure, lack of management guidelines and poor hygiene [17, 20]. Other problems include health workforce challenges like poor qualification of health care providers, uneven distribution and rapid turnover of the health workforce and lack of continuing educational and professional development opportunities; and dearth of resources including shortage and low quality of medical supplies and inadequate financing [21]. Poor information technology and poor leadership are also major obstacles to the antenatal care [22]. This situation of health care sector in Iraq may affect the use of CAM among pregnant women.

The present study was planned to gain insights into the prevalence and factors leading to the use of CAM among pregnant women in Iraq. Additionally, the study was designed to bring to light CAM therapies which are most commonly used during pregnancy in the gulf country. The study was also purposely designed to identify the main sources of information recommending the use of CAM during pregnancy.

## Methods

For this study, a cross-sectional survey was designed to collect information on the use of CAM among Iraqi pregnant women. The data was gathered in outpatient department of four hospitals located in Basrah, Iraq. Eligible participants of the study were Iraqi women who visited outpatient department to seek antenatal care services. Women who visited the hospitals for normal delivery, cesarean section or post-natal checkup were excluded from the study.

The survey questionnaire was designed in the English language and then translated into the Arabic language to best suit the target population. The Arabic version of questionnaire was validated by translation back into English, and the text was revised where necessary by three doctors who were not among authors of this study. Then it was piloted on a small group of 20 volunteers. In order to collect the information, one week training was given to eight surveyors mainly on ethics and data collection. Two surveyors were asked to gather data from each hospital. Before the interview, surveyors explained nature of the study to the participants and sought consent. The surveyors conducted the face-to-face interviews with the participants and fill out the questionnaire during the period of four weeks from November 13, 2014 to December 15, 2014.

The survey questionnaire comprised of 34 items, close and open-ended questions, divided into three sections: demographic characteristics, medical factors and questions on CAM related information. CAM related questions were asked to collect information on modalities of CAM used and frequency of CAM use, reasons for using CAM, satisfaction with usage, disclosure of CAM, supplier of CAM and source of CAM information. The collected data were coded and analyzed using Statistical Package for Social Sciences (SPSS) v. 21. To analyze categorical variables related to CAM use, perceptions and attitudes, descriptive statistics (using n and percentage) were used. In order to see the relationship between CAM use and characteristics of the survey participants, chi-square test and logistic regression analysis were done considering *P* value <0.05 to be statistically significant.

## Results

### Participant characteristics

The characteristics of the study participants are shown in Table 1. A total of 335 pregnant women were consecutively interviewed, with response rate of 83.96 %. The mean age was 26.1 ± 6.9, 74.6 % were aged ≤30 years, 59.7 % were living in urban areas, 89.3 % were housewife, 42.1 % had at least a middle school education and 35.8 % had household income ≤500,000 Iraqi Dirhams. In terms of medical characteristics, 45.1 % perceived themselves as healthy, 35.5 % had history of previous pregnancy, and 31.9 % used contraception ever.

**Table 1:** Demographic and health related characteristics of participants (*N*=335)

Variables		CAM Users (%)	Non users (%)	Total	*X* ^*2*^	*P value*
Age	20 years and below	52 (27.4)	39 (26.9)	91	0.010	0.995
	21–30 years	90 (47.4)	69 (47.6)	159		
	31-41years	48 (25.3)	37 (25.5)	85		
Residence	Urban	102 (53.7)	98 (67.6)	200	6.606	0.013*
	Rural	88 (46.3)	47 (32.4)	135		
Occupation	Yes	12 (6.3)	24 (16.6)	36	8.984	0.003**
	No(housewife)	178 (93.7)	121 (83.4)	299		
Education level	No schooling	39 (20.5)	36 (24.8)	75	1.101	0.577
	Elementary school	71 (37.4)	48 (33.1)	119		
	Middle school or higher	80 (42.1)	61 (42.1)	141		
Monthly income	<500,000 ID^+^	48 (25.3)	72 (49.7)	120	21.284	0.000***
	500,001–800,000 ID	76 (40.0)	39 (26.9)	115		
	>800,001 ID	66 (34.7)	34 (23.4)	100		
Perceived health status	Unhealthy	39 (20.5)	73 (50.3)	112	34.033	0.000***
	Average	45 (23.7)	27 (18.6)	72		
	Healthy	106 (55.8)	45 (31.0)	151		
Previous history of pregnancy	No	118 (62.1)	98 (67.6)	216	1.079	0.299
	Yes	72 (37.9)	47 (32.4)	119		
Use of Contraception	No	98 (51.6)	130 (89.7)	228	54.847	0.000***
	Yes	92 (48.4)	15 (10.3)	107		

^+^One US Dollar equaled about 1,175 Iraqi Dinars during time of study

*RC =* Reference category

*p Value <0.05

**p Value <0.01

***p Value <0.001

### Utilization of CAM

Table 1 illustrates utilization of CAM among study participants. In total 56.7 % of the pregnant women reported using CAM. 47.4 % of the CAM users aged 21–30 years, 53.7 % were living in urban areas, 93.7 % were housewife, 42.1 % had at least a middle school education, 40 % were from middle income group, 55.8 % perceived themselves healthy, 37.9 % had history of previous pregnancy, and 51.6 % never used contraception.

### Factors affecting CAM use

Chi-square test revealed that residence of living, occupation, monthly income, perceived health status and ever use of contraception were associated with use of CAM (Table 1). Results of logistic regression analysis showing factors affecting the use of CAM are presented in Table 2. Rural residence of living (*p*<0.01), no occupation (*p*<0.05), higher monthly income (*p*<0.05), perceived healthy status (*p*<0.05), and ever use of contraception (*p*<0.01) were positively associated with the utilization of CAM.

**Table 2:** Logistic regression analysis for determining factors affecting CAM use

Characteristic	Group	OR	95 % CI		*p* Value
Residence	Urban				*RC*
	Rural	2.015	1.194	3.401	0.009**
Occupation	Yes				*RC*
	No (Housewife)	2.749	1.161	6.512	0.022*
Education level	No schooling				*RC*
	Elementary school	1.261	0.634	2.510	0.508
	Middle school or higher	1.729	0.855	3.496	0.127
Monthly income	<500,000 ID				*RC*
	500,001–800,000 ID	1.910	1.032	3.555	0.039*
	>800,001 ID	2.060	1.055	4.033	0.034*
Perceived health status	Unhealthy				*RC*
	Average	2.608	1.346	5.056	.005**
	Healthy	2.595	1.255	5.365	.010*
Previous history of pregnancy	No				*RC*
	Yes	1.272	0.807	2.005	0.299
Use of contraception	No				*RC*
	Yes	2.039	1.249	3.329	0.004**

*RC =* Reference category

*p Value <0.05

**p Value <0.01

### Frequency and modalities of CAM use

**Fig. 1:**
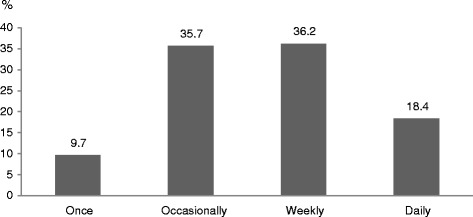
Frequency of CAM use during current pregnancy

Figure 1 displays the frequency of CAM use among study participants. In total, 36.2 % used CAM twice a week, 35.7 % sometimes, 18.4 % daily and 9.7 % had used only once. Table 3 illustrates types of CAM modalities used by the pregnant women. Among CAM users, 53.7 % reported using herbs or natural products and 36.3 % used vitamins. Black seed (16.5 %), chamomile (16.2 %), cinnamon (10.8 %), castor oil plant (9.4 %) and ginger (8.3 %) were the most popular natural products.

**Table 3:** Frequencies of complementary and alternative medicine (CAM) use by type

Types of CAM	No. of Users	Percent
Vitamins	69	36.3
Massage	9	4.7
Exercise	6	3.2
Energy therapy	4	2.1
Herbs or natural products		53.7
Black seed	58	16.5
Chamomile	57	16.2
Cinnamon	38	10.8
Castor oil plant	33	9.4
Ginger	29	8.3
Royal honey	21	6
Garlic	19	5.4
Peppermint	14	4
Olive oil	11	3.1
Unidentified mixed spices	10	2.8
Cumin	8	2.3
Licorice	8	2.3
Thyme	4	1.1
Eucalyptus leaves	3	0.9
Green tea	3	0.9
Special type of spices	2	0.6
Other types of CAM		7.2
Metal ring	21	6
Medicinal talisman & amulet	8	2.3
Gemstones	2	0.6
Precious stones	1	0.3

### Reason for using CAM and satisfaction

Reasons for using CAM and CAM satisfaction are shown in Table 4. About 43 % of the CAM users considered that it was not dangerous for their pregnancy, 28.6 % used for its effectiveness, 17.5 % for cultural reasons, and 10.6 % for the safety of fetus. Moreover, 83.7 % were satisfied with their CAM use.

**Table 4:** Reasons for using CAM and satisfaction with CAM

Variable	Number	Percent
Why use CAM (*N* = 189)		
Not dangerous for pregnancy	81	42.9
Effectiveness	54	28.6
Cultural reasons for using herbs	33	17.5
Safety of fetus	20	10.6
Accessibility	1	0.5
Satisfaction (*N* = 190)		
Satisfied	159	83.7
Unsatisfied	31	16.3

### Disclosure of CAM use

Table 5 illustrates CAM users’ disclosure of CAM to their doctors. In total only 0.5 % discussed their use of CAM with doctor. Reasons for the non-disclosure were: doctors did not ask 50.53 %, 28.2 % did not consider it important to disclose and 5.3 % were afraid of doctor’s response.

**Table 5:** Disclosure of CAM use to and reasons for non-disclosure to a medical doctor

Variables	Number	Percent
Discuss with doctor (*N* = 190)		
Yes	1	0.5
No	189	99.5
Reasons for non-disclosure (*N* = 188)		
Doctor does not ask	95	50.5
Not important to talk	53	28.2
Got treated with CAM even before pregnancy	17	9.1
No specific reason	13	6.9
Afraid of doctor’s response	10	5.3

### Source of information on CAM and supplier of CAM

Source of information on CAM and supplier of CAM are displayed in Table 6. For 46.3 % source of CAM information was friend, family 18.4 % and neighbor 16.3 %. In total, 63.8 % got CAM from shops and 33 % from CAM providers.

**Table 6:** Source of information on and supplier of CAM

Variables		Number	Percent
Source of Information on CAM (*N* = 190)	Friends	88	46.3
	Family	35	18.4
	Neighbor	31	16.3
	Newspaper, magazine	16	8.4
	Television, radio	12	6.3
	Religious group	6	1.8
	Health professionals	2	1.1
Supplier of CAM (*N* = 185)	Market/shop	118	63.8
	CAM provider	61	33.0
	Family	5	2.7
	Religious group	1	0.5

## Discussion

This is the first study investigating knowledge, attitude and practice of CAM among pregnant women in Iraq, and is also the first work conducted with the aim of describing CAM users. The findings emphasize that the use of CAM during pregnancy is a common habit in the city of Basrah, Iraq. The study followed cross-sectional design and gathered information on the use of CAM by pregnant Iraqi women who visited outpatient department of four hospitals located in Basrah.

We found that 56.7 % of Iraqi women use at least one modality of CAM during pregnancy. Our results suggest significantly higher use of CAM among pregnant women in Iraq when compared to similar studies conducted in India, Oman, Zimbabwe, Palestine, Malaysia, Egypt, and Taiwan [10, 23–28]. However, it is lower than the studies done in Iran, Jordan, and UK [6, 11, 29]. These variations in the prevalence of CAM are understandable as difference in the proportion of CAM use could come from a number of factors, such as design of study, sample dynamics, and socio-demographic factors. This study also highlighted the extremely low level of communication, regarding use of CAM, between patients and their healthcare providers.

Likely predictors of CAM use among pregnant women in this study were residential area, occupation status and monthly income, which is consistent with previous studies [11, 25, 26]. We also found that pregnant women who perceived their health status as healthy tended to use CAM more than women who perceived their health status average or unhealthy. It makes sense as evidence suggests that pregnant women start using CAM, or more specifically herbs, in order to enhance their health status, increasing immunity, strengthening uterus ready for the labor and to avoid complications related to pregnancy like urinary tract infections and digestive disorders [29, 30]. Therefore, it is possible that pregnant women in this study did not perceive themselves healthy before using CAM; but once they started to use CAM, they felt more healthy and prepared for the labor. Furthermore, we demonstrated that women who ever used contraception were also more likely to be the CAM users, which is supported by the study conducted in Zambia [31]. In our study, more than 50 % of the pregnant women used herbal medication as a CAM modality. There is ample evidence suggesting high consumption of herbs, and that herbal products are the most common form of CAM, among pregnant women due to several reasons [32]. These studies suggest pregnant women use herbal supplements, such as raspberry leaf, ginger, chamomile, and cranberry juice, to gain strength in order to get prepared for the labor [30, 33]. The second most commonly used CAM modality among pregnant women was consumption of vitamins, which is also consistent with previous research [23, 24, 34]. We found that most of the CAM users were satisfied with the CAM used; and for 28 % of them effectiveness of the CAM use was main reason, as the most common reason for consumption was given as “not dangerous for pregnancy”. We also discovered that only about 18 % of the CAM users consumed it on daily basis while most of them utilized herbal products and vitamins for not more than twice a week. Our results reveal that about 64 % of the CAM users could get CAM from marketplace, which highlights the easy availability of CAM, especially herbal products, in the region. Additionally, our results show that most common source of CAM information was friends and family, which is supported by the previous study conducted by Holst et al. [35].

CAM users in our sample used various herbal therapies during pregnancy. The most popular herbal therapy was consumption of black seed and chamomile. Al-Riyami et al. report that pregnant women could use black seed during pregnancy against infections or as nutritional supplement while chamomile as a relaxant [23]. Literature suggest that herbal products could be harmful to the mother and fetus if consumed during pregnancy because herbs contain pharmacologically active substances [36]. Herbal supplements like garlic, ginger and chamomile have anti-platelet and anticoagulant properties so there is an increased risk of prolonged clotting time and bleeding [37]. Hypertensive and hyperglycemic characteristics of licorice may increase the complications of pre-eclampsia and gestational diabetes respectively [38]. Moreover, there is also an increased risk of preterm labor and miscarriage among regular users of chamomile and licorice [39]. However, pregnant women consider herbs safer than conventional medications due to their extensive experience with and belief in herbal products [40]. Many pregnant users of CAM are unaware of their side-effects because they consider it natural and risk-free [37, 41].

We also discovered extremely low proportion of CAM users disclosing their CAM use to the doctors – only 0.5 % did so. More than 50 % of the respondents did not discuss their use of CAM because their doctors did not ask them. Moreover, the CAM users did not think it was important to discuss with their health providers. Poor communication regarding CAM between patients and doctors in our sample could be attributed to the CAM users’ unawareness of adverse effects from alternative medications as only 42 % of them had middle school education. Therefore, it highlights the need to increase awareness of Iraqi women on the safe use of CAM during pregnancy. It is also crucial that health care providers should have basic understanding about CAM including herbal products, so that they can better recognize the risks associated with health seeking behavior of patients [42]. Additionally, we recommend that physicians, as qualified healthcare providers, should ensure good level of communication with patients for their safety.

### Limitations

This study has some limitations. Firstly, a bigger sample size would have been preferred if there were no constraints on time and resources. Nevertheless, we did our best by generating sample from four public hospitals of Basrah city. However, it does not represent the national population; therefore, the results cannot be applied to all Iraqi women. Our sample is also harmonized in terms of socio-demographic factors except for the occupation. Secondly, this study did not include individual benefits of each modality of CAM used by pregnant women. Instead, we focused on attitudes of CAM users towards CAM and conventional healthcare.

## Conclusions

Results of the study underline that use of CAM is common during pregnancy in Iraq. In total, twenty-four different modalities of CAM were used for pregnancy-related health ailments, most frequently vitamins, black seed, chamomile and cinnamon. Moreover, extremely low level of communication between CAM users and their health care providers is worrisome, and demands that physicians should inquire their patients about the use of CAM. Due to scarcity of evidence in support of CAM benefits during pregnancy, it is very important to educate women on the safe use of CAM especially when pregnant women come from a low-literate population such as our sample. Based on the results of our research, we recommend that this area should further be studied using larger sample size.

## Abbreviation

CAM, complementary and alternative medicine
